# Mechanotransduction in Marfan Syndrome and Related Aortic Disorders: Insights from Transcriptomic Analyses

**DOI:** 10.3390/genes17070770

**Published:** 2026-06-30

**Authors:** Anna Cantalupo, Jason R. Cook, Jens Hansen, Samia Lasaad, Lisa M. Satlin, Ravi Iyengar

**Affiliations:** 1 Department of Pediatrics, Icahn School of Medicine at Mount Sinai, New York, NY 10029, USA; samia.lasaad@mssm.edu (S.L.); lisa.satlin@mssm.edu (L.M.S.); 2 Division of Vascular Surgery, Department of Surgery, University of Nebraska Medical Center, Omaha, NE 68198, USA; jason.cook@unmc.edu; 3 Department of Pharmacological Sciences, Icahn School of Medicine at Mount Sinai, New York, NY 10029, USA; jens.hansen@mssm.edu (J.H.); ravi.iyengar@mssm.edu (R.I.)

**Keywords:** heritable thoracic aortic diseases, Marfan syndrome, mechanotransduction, transcriptomic analyses

## Abstract

Heritable thoracic aortic diseases (HTADs) comprise a genetically heterogeneous group of disorders predisposing patients to thoracic aortic aneurysm and dissection, yet current medical therapies remain limited to slowing disease progression rather than preventing aortic wall failure. Although pathogenic variants affect diverse genes encoding extracellular matrix (ECM) components, smooth muscle contractile proteins, and signaling molecules, these defects converge on disruption of the mechanobiological systems that maintain aortic wall integrity. The thoracic aorta functions as a mechanically integrated tissue in which endothelial cells, vascular smooth muscle cells, fibroblasts, immune cells and ECM continuously sense and respond to pulsatile biomechanical forces. Genetic perturbations affecting ECM architecture, contractile force generation, or growth factor signaling alter force transmission across this multicellular network, leading to maladaptive mechanotransduction, cellular phenotypic modulation, and progressive aneurysm formation. Using Marfan syndrome as a paradigmatic ECM-driven aortic disease, this review synthesizes current understanding of how altered biomechanics, biochemical signaling and immune responses reshape intercellular communication and activate disease-associated signaling pathways, including dysregulated TGF-β, nitric oxide, angiotensin receptor, calcium-dependent, and metabolic signaling. We highlight how single-cell transcriptomic analyses have elaborated changes in different cell-level functions including, ECM degradation, iron homeostasis, circadian/stress responses. Changes in iron metabolism in different cell types in the aorta suggest possible coordinated metabolic changes in aneurysm progression. These mechanistic insights enable the identification of cell-type–specific pathogenic programs and therapeutic discovery through systems-level approaches. We highlight the translational opportunities and challenges emerging from mouse models and human studies, emphasizing that therapeutic efficacy depends not only on pathway selection but also on disease stage, cellular context, and timing of intervention. Together, these findings support a model in which HTAD progression reflects dynamic, multicellular failure of mechanobiological homeostasis and provide a framework for the development of more precise, mechanism-based therapies.

## 1. Introduction

Heritable thoracic aortic diseases (HTADs) comprise a genetically heterogeneous group of disorders that predispose individuals to thoracic aortic aneurysm and dissection, often in the absence of early clinical symptoms [1,2]. Despite advances in genetic diagnosis and surveillance imaging, prophylactic surgery remains the primary life-saving intervention. Currently available pharmacologic therapies slow disease progression in some patients but do not prevent aneurysm formation or dissection [1,2,3,4]. This clinical reality underscores the need to better understand the molecular and cellular mechanisms that drive aortic wall failure, so that more effective drugs can be developed to block aneurysm progression and prevent dissection.

HTAD is primarily a biomechanical disease. Pathogenic variants cluster in genes encoding extracellular matrix (ECM) components [5,6,7], vascular smooth muscle contractile proteins [1,8,9], and regulators of transforming growth factor-β (TGF-β) signaling [10,11]. Marfan syndrome, caused by mutations in *FBN1*, represents the prototypical ECM-driven aortic disease, in which disruption of fibrillin-1 microfibrils alters elastic fiber architecture, growth factor bioavailability, and the mechanical integrity of the aortic wall [5,12]. These structural alterations primarily affect the medial layer, where vascular smooth muscle cells (VSMCs) interact with elastic fibers to maintain contractile tone and structural resilience. In parallel, mutations in contractile genes such as *ACTA2* and *MYH11* directly impair VSMC force generation and cytoskeletal organization [13,14]. At the luminal interface, endothelial cells (ECs) sense hemodynamic forces and regulate vascular tone, inflammation, and redox balance [15,16]. Alterations in TGF-β signaling and ECM architecture can disrupt endothelial–smooth muscle communication and modify cellular responses to mechanical cues [17]. Thus, genetic defects affecting ECM structure, contractile machinery, and signaling pathways converge to disrupt the coordinated mechanobiological functions of the endothelial and medial compartments required for aortic homeostasis, ultimately predisposing the vessel wall to aneurysm formation.

Despite this genetic diversity, these defects converge on a common physiological vulnerability: impaired mechanotransduction. The aorta is a dynamic organ that continually senses mechanical forces, including blood flow-mediated shear stress and blood pressure-associated circumferential and axial strain [18], which are transduced into adaptative cellular responses. This integrated sensing and adaptation is essential for vascular homeostasis. Disruption of the generation, detection, transmission, or interpretation of biomechanical forces compromises these adaptive responses, promoting maladaptive remodeling and progressive dilation [2]. Understanding how genetic alterations in ECM components and cellular contractile machinery reshape these mechanobiological processes is therefore critical for defining the molecular and cellular basis of aortic disease.

In this review, we discuss the genetic architecture of HTAD with emphasis on ECM-associated genes, examine how defects in ECM structure and cellular mechanotransduction disrupt communication between the endothelial and smooth muscle compartments of the aortic wall, and highlight emerging molecular pathways that may represent therapeutic targets. We focus on Marfan syndrome as a paradigmatic ECM-driven aortopathy, providing a framework to integrate insights from biomechanics, vascular cell biology, transcriptomics, and translational studies aimed at stabilizing the aortic wall before dissection occurs. We describe recent advances in mechanobiology and single-cell transcriptomics that provide new insights into the complex cellular processes involved in HTAD. These studies have revealed previously unrecognized cellular heterogeneity, disease-associated cell states, and potential dynamic interactions among ECs, VSMCs, fibroblasts, and immune populations. By integrating genetic, transcriptomic, and functional studies, we discuss how defects in ECM architecture, mechanosensing, cellular metabolism, and intercellular communication converge to drive aneurysm progression across the intimal, medial, and adventitial compartments of the aortic wall. We also highlight how these emerging insights may inform the development of stage-specific and cell-targeted therapeutic strategies.

## 2. Cellular and Extracellular Architecture of the Aortic Wall in Health and Disease

**Figure 1:**
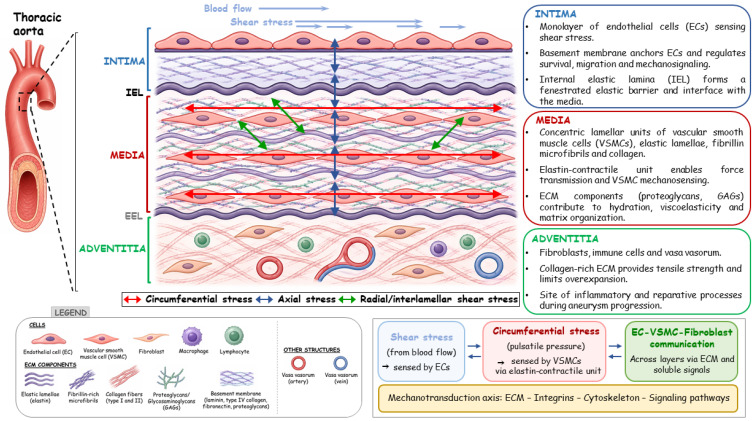
Schematic representation of the thoracic aortic wall. The figure illustrates the structural organization of the intimal, medial, and adventitial layers and the major cellular and extracellular matrix components that coordinate force transmission, mechanotransduction, and vascular homeostasis.

The thoracic aorta is organized into three interconnected layers—intima, media, and adventitia—in which vascular cells are embedded within an ECM that provides structural integrity and enables mechanosensitive signaling under pulsatile load. The intima consists of ECs that sense hemodynamic forces and regulate vascular tone and inflammation. The media contains VSMCs arranged within elastic lamellar units that support force transmission and adaptive remodeling. The adventitia is enriched in fibroblasts and collagen, contributing to tensile strength and serving as a site of immune and reparative responses. The structural and functional interactions among these layers are summarized in Figure 1.

ECM assembly within the aortic wall is dynamic and spatially regulated in response to mechanical and biochemical cues [19]. Although VSMCs have traditionally been considered the primary source of ECM proteins within the arterial wall, recent evidence challenges this view. Lineage-tracing studies of elastin have demonstrated that VSMCs generate medial elastic lamellae in elastic arteries such as the aorta, whereas ECs can contribute to elastin within the internal elastic lamina (IEL) in smaller arteries [20]. This organization implies that mechanical cues differ across layers and vascular beds. Beyond its structural role, ECM functions as a dynamic signaling platform that regulates cellular behavior through interactions with cell surface receptors and growth factor reservoirs, thereby modulating processes such as cell survival, differentiation, and inflammatory signaling. Accordingly, disruption of this tightly coordinated cellular–ECM system is a defining feature of thoracic aortic aneurysm, in which altered matrix organization, defective force transmission, and dysregulated signaling progressively destabilize the vessel wall [1,2,21].

Marfan syndrome exemplifies how defects in ECM architecture can destabilize this system. Mutations in *FBN1* impair fibrillin-1 microfibril formation, disrupt elastic fiber organization, and alter TGF-β regulation, thereby compromising the ability of the wall to sense, transmit, and buffer mechanical stress [5,22,23]. This does not simply weaken the aortic wall; it changes how resident cells interpret their physical environment, promoting maladaptive remodeling and aneurysm progression. Recent biomechanical studies further suggest that aneurysm progression and susceptibility to dissection or rupture may reflect multiple distinct pathological processes. Using direct measurements of aortic rupture force across multiple hereditary aortopathy mouse models, Dubacher et al. compared defects affecting fibrillin-1 (*FBN1*), fibulin-4 (*EFEMP2*), latent TGF-β binding protein-1 (*LTBP1*), microfibril-associated glycoprotein-4 (*Mfap4*), and tissue inhibitor of metalloproteinases-1 (*TIMP1*) [24]. While *FBN1* and *EFEM2* defects significantly impaired biomechanical integrity, *LTBP1*, *MFAP4*, and *TIMP1* variants showed preserved aortic rupture force. Notably, losartan treatment reduced aneurysm formation in *Fbn1^mgR/mgR^* mice but failed to improve aortic rupture force, indicating that attenuation of aortic enlargement does not necessarily restore the mechanical competence of the vessel wall [24]. Together, these findings suggest that the determinants of aneurysm growth, wall integrity, and rupture risk may not completely overlap and highlight the diverse functional contributions of individual ECM components to aortic homeostasis.

Recent advances in single-cell transcriptomic technologies have substantially extended our understanding of the dynamic cellular composition and functional states of the aortic wall in health and disease. Single-cell/single nucleus RNA sequencing (sc/snRNA-seq) studies have revealed that major vascular cell types, including ECs and VSMCs, are not homogeneous populations but comprise multiple transcriptionally distinct subpopulations with specialized roles in matrix production, contractility, inflammation, and mechanosensing [25,26]. For example, VSMCs exist along a spectrum ranging from contractile cells to modulated states characterized by reduced expression of cytoskeletal genes and increased expression of ECM and signaling molecules [27,28], while ECs display functionally distinct subsets associated with barrier function, angiogenesis, and flow-responsive signaling [26,29,30].

Importantly, single-cell analyses of aneurysmal aortas have identified disease-specific cell states not present under physiological conditions. In Marfan syndrome, both mouse and human studies have described transcriptionally modulated smooth muscle cell populations associated with aneurysm development, enriched in pathways related to ECM remodeling and cell–matrix interactions [31,32,33]. These disease-associated responses are influenced by developmental origin. Work from MacFarlane et al. in Loeys–Dietz syndrome established that neural crest– and second heart field–derived smooth muscle populations have distinct susceptibilities to aneurysm formation at the aortic root [34], and Pedroza et al. extended this concept to Marfan syndrome by demonstrating lineage-specific smooth muscle phenotypic modulation signatures in the murine Marfan aorta [35]. These findings identify developmental lineage as an important determinant of disease-associated cellular behavior. Although murine models of Marfan syndrome range in disease severity, from the severe *Fbn1*^mgR/mgR^ model to the milder *Fbn1*^C1041G/+^ model, single-cell studies across these systems consistently identify similar modulated VSMC states and altered endothelial programs, supporting the robustness of these transcriptional signatures. In our recent work, scRNA-seq profiling of the dissecting aorta in *Fbn1*^mgR/mgR^ mice identified a discrete disease-associated cell population (MFSmod), arising from closely related endothelial and smooth muscle subclusters (EC4 and SMC3), characterized by increased expression of ECM components, nitric oxide (NO)–related genes, and markers of altered cell identity [33]. These cells are localized to the intimal region of the diseased aorta and exhibit strong transcriptomic similarity to modulated smooth muscle cell populations identified in both mouse models and human thoracic aortic aneurysm tissue [31,32].

Collectively, these findings indicate that aneurysm formation is associated with dynamic changes in cell identity and intercellular signaling, rather than static alterations in cell composition. The emergence of modulated cell states reflects coordinated alterations in pathways regulating cytoskeletal organization, ECM remodeling, and cell–matrix communication, pointing to disrupted mechanotransduction as a central driver of disease-associated cellular reprogramming across the aortic wall. These studies also support a view of the aorta as a multicellular, mechanically integrated tissue in which ECM composition, developmental origin, and cell-state transitions interact to determine regional susceptibility to aneurysm. Transcriptomic approaches have provided not only mechanistic insight but also clinically relevant direction, by identifying pathway-level changes and disease-associated cell populations that can be prioritized for functional validation and therapeutic targeting. These mechanistic insights relate directly to the underlying genetic architecture of HTAD, in which variants affecting ECM structure, cytoskeletal function, and intracellular signaling pathways converge on a limited set of cellular processes (Table 1), discussed in detail in Section 7.

## 3. Cell-Type-Specific Changes in Transcriptional Programs in Aneurysm Development

Comparative analysis of single-cell and single-nucleus transcriptomic datasets from human thoracic aortic aneurysm tissue [36] and Marfan (MFS) mouse aorta [33], using complementary Gene Ontology and Molecular Biology of the Cell Ontology approaches, provides insight into conserved and divergent transcriptional programs across vascular cell types during aneurysm progression. Although direct comparison is limited by differences in disease stage—human samples representing advanced aneurysms and mouse samples reflecting earlier-stage disease—several informative parallels and distinctions emerge.

Across species, ECM dysregulation and altered vascular remodeling programs are shared features of aneurysm development, supporting the concept that matrix remodeling is a common hallmark of disease. However, major differences are evident in the transcriptional state of individual cell populations.

In VSMCs, we identify dysregulated ECM dynamics, cell–matrix adhesion and actin cytoskeletal dynamics in MFS mice and human aneurysm, potentially linked to the predicted blood vessel developmental pathways. Predicted dysregulation in LINC-complex mediated nucleo-cytoskeletal coupling in human VSMCs has been linked to a rare genetic variant associated with inherited thoracic aneurysms [37]. Human VSMCs document downregulation of antioxidant defense pathways, in agreement with previous findings [38]. MFS mice VSMCs display downregulation of mitochondrial energy production, ribosomal translation and muscle contraction. The latter is the transcriptional target of baclofen, a drug that we identified using mouse and human bulk transcriptomics and that delays aneurysm progression in MFS mice [39].

Analysis of gene expression patterns in ECs in human aneurysms suggests endothelial migration and angiogenesis, dysregulation in leukocyte adhesion and transmigration, ECM degradation, and apoptosis. Mouse ECs show upregulation of pathways involved in humoral immune response, e.g., complement, and downregulation of pathways involved in translation.

MFS and human fibroblast analyses indicate ECM secretion, remodeling activities and vessel morphogenesis activities. Additionally, and similarly to human VSMCs, human fibroblasts downregulate antioxidant defense pathways.

Human aneurysm macrophages displays immune-related signaling and potential ECM-degradation activities. In contrast, enrichment analysis for MFS mice macrophages suggests proliferation.

**Figure 2:**
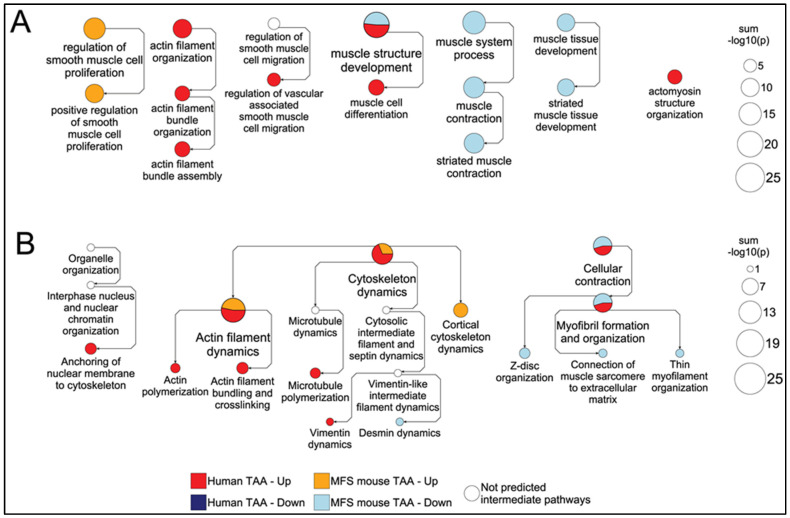
Aneurysm-associated processes in human and MFS mice VSMCs. Genes that are up- and downregulated in disease vs. control VSMCs were subjected to pathway enrichment analysis using (**A**) Gene Ontology Biological Processes and (**B**) the Molecular Biology of the Cell Ontology. Predicted processes were integrated in related parent–child hierarchies. Selected branches involved in contraction and cytoskeletal dynamics are shown. Pie slice areas represent significance.

Considering all four cell types together, several patterns are identified across the two ontologies, with four major features of the human disease emerging. First, inflammatory engagement may be a human-selective feature, with macrophages as the central orchestrator [33,36]. Second, ECM degradation occurs in multiple vascular cells, in which macrophages (heparanases/sulfatases), fibroblasts (ADAMalysins), and ECs (ADAMalysins) all contribute. This implies that aneurysm matrix breakdown is a coordinated multi-cell-type process, not just a fibroblast/VSMC phenomenon [33,36]. Iron homeostasis is suppressed across VSMCs, ECs, and fibroblasts [36], suggesting a potential unifying metabolic phenotype. The relevance of iron metabolism in multiple cell types involved in aortic aneurysms has been recently reviewed [40]. Although effects of iron metabolism on individual cell types are readily discernable, we do not as yet understand if there is coordinated regulation between the different cell types and what if any such coordination plays in contributing to aortic aneurysm states. Lastly, circadian and stress-hormone responses are upregulated in humans across fibroblasts and macrophages, suggesting a systemic neuroendocrine/circadian dimension to human aneurysms. An example of the differences between human and mouse features from the sc/snRNA-seq experiments for VSMC contractility is shown in Figure 2.

Together, these findings indicate that mouse models reproduce many early transcriptional features of aneurysm development but incompletely capture the chronic inflammatory and degenerative programs observed in advanced human disease. These differences should be considered when extrapolating mechanistic and therapeutic insights from mouse models to human HTAD.

## 4. Mechanotransduction Pathways in Aortic Wall Cells

The thoracic aorta is continuously exposed to pulsatile mechanical forces that must be sensed and translated into coordinated cellular responses to preserve wall integrity. Mechanotransduction integrates ECM architecture, cell–matrix adhesion, cytoskeletal tension, and intracellular signaling pathways across endothelial and smooth muscle compartments [41]. In HTAD, these pathways can be viewed as components of an interconnected mechanosensitive signaling network in which abnormalities in ECM structure and biomechanics influence how ECs and VSMCs sense and respond to wall stress.

**Figure 3:**
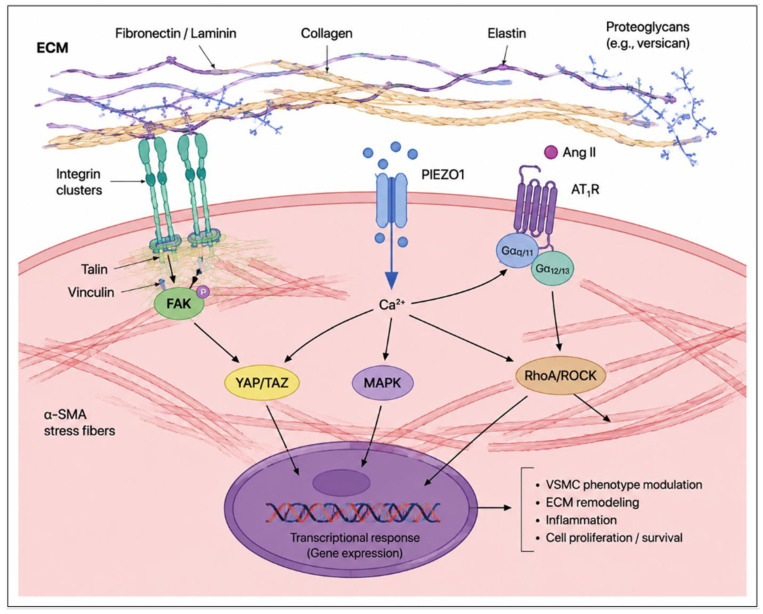
Major mechanotransduction pathways implicated in HTAD. Extracellular matrix-derived mechanical and biomechanical signals are sensed by vascular cells through multiple mechanotransduction pathways, leading to activation of intracellular signaling networks and transcriptional programs involved in vascular adaptation, remodeling, and disease progression.

Fibrillin-rich microfibrils and elastic lamellae provide not only structural support but also regulate how vascular cells perceive and respond to biomechanical stress by modulating both force transmission and growth factor bioavailability [42]. Mechanical forces are transmitted to VSMCs through the elastin–integrin–cytoskeleton axis, in which ECM structures are physically coupled to focal adhesions and the actomyosin contractile apparatus [21,43]. This organization translates wall tension into intracellular signals that regulate contractility, calcium handling, gene expression, and maintenance of the contractile phenotype [8,21,44,45] (Figure 3). 

Among the pathways linking ECM mechanics to cellular responses, YAP/TAZ signaling has emerged as a central mechanosensitive hub. Changes in ECM stiffness and cytoskeletal tension regulate YAP/TAZ activity, allowing vascular cells to translate biomechanical cues into transcriptional programs that control contractility, cytoskeletal organization, and adaptive remodeling [46,47]. In HTAD, mutations affecting ECM structure (*FBN1*) or contractile machinery (*ACTA2*, *MYH11*) disrupt this force-transmission system, impairing the ability of VSMCs to appropriately sense and adapt to wall stress and promoting maladaptive remodeling [1,8].

TGF-β signaling represents a central mechanosensitive pathway linking ECM structure to cellular responses. Matrix tension regulates activation of latent TGF-β complexes through integrin-dependent mechanisms, coupling mechanical stress to signaling output [23]. Basal TGF-β signaling is required for postnatal aortic growth and smooth muscle homeostasis, indicating that this pathway serves physiological as well as pathological functions. Accordingly, fibrillin-1 does not simply provide structural support but also helps determine how vascular cells interpret mechanical stress through regulation of TGF-β bioavailability and signaling [42]. Disruption of this system in HTAD alters both the magnitude and spatial distribution of TGF-β signaling. Emerging evidence suggests that the consequences of altered TGF-β activity extend beyond transcriptional regulation and ECM remodeling to include cellular metabolism. Studies in Marfan syndrome mice demonstrated that ECM abnormalities are associated with impaired mitochondrial respiration and altered NAD metabolism, linking defects in the extracellular environment to metabolic dysfunction within the aortic wall [48]. Importantly, restoration of mitochondrial metabolism through supplementation with the NAD precursor nicotinamide riboside reversed established aortic aneurysm in *Fbn1^C1041G/+^* mice, supporting a functional contribution of metabolic dysfunction to disease progression rather than a simple secondary consequence of tissue injury. Consistent with these observations, mitochondrial dysfunction and impaired oxidative phosphorylation are increasingly recognized in aneurysmal tissue, and may contribute to disease progression [49,50]. Recent work has further expanded the functional repertoire of the ECM by demonstrating a direct role in the regulation of mitochondrial homeostasis and cellular metabolism [51]. Although this study was not performed in the context of aneurysm disease, it provides a conceptual framework through which ECM abnormalities could influence mitochondrial function independently of their established effects on force transmission and growth factor signaling. Together with observations from Marfan syndrome models showing impaired mitochondrial respiration and responsiveness to nicotinamide riboside, these findings support the emerging view that ECM integrity, mechanotransduction, and cellular metabolism are tightly interconnected processes. Beyond their effects on cellular metabolism, alterations in TGF-β signaling also influence communication between vascular cell populations. Importantly, smooth muscle–specific loss of TGF-β signaling induces endothelial dysfunction, which in turn contributes to aortic hypercontractility, highlighting a critical axis of communication between the medial and intimal compartments [17]. These findings, together with sc/snRNA-seq studies, emphasize that mechanotransduction defects in HTAD are not confined to a single cell type but propagate across the vessel wall. Thus, disruption of ECM architecture and force transmission can alter TGF-β bioavailability and signaling, with downstream effects on endothelial function, VSMC phenotype and contractility, and communication between the intimal and medial compartments.

Multiple mechanosensitive pathways contribute to the ability of vascular cells to sense and respond to changes in blood flow, wall tension, and ECM architecture. Rather than operating independently, these pathways form an interconnected network that integrates biomechanical inputs into transcriptional, metabolic, and functional responses. The following examples illustrate several of the best-characterized mechanotransduction systems implicated in HTAD pathogenesis.

ECs form the principal flow-sensing layer of the aortic wall. At endothelial junctions, PECAM1, VE-cadherin, and VEGFR2 form a mechanosensory complex that transduces shear stress into intracellular signaling [52]. These inputs promote eNOS activation and nitric oxide (NO) production, induce transcription factors such as KLF2 and KLF4, and suppress inflammatory signaling to maintain endothelial quiescence [53,54,55]. YAP and TAZ are mechanosensitive transcriptional co-activators that integrate biomechanical cues from the ECM, cytoskeleton, and cell–cell junctions into adaptive gene-expression programs [46,56,57]. Unlike many signaling mediators that operate downstream of specific receptors, YAP/TAZ can directly respond to changes in substrate stiffness, actomyosin tension, cell shape, and shear stress, making them central regulators of cellular mechanotransduction. In ECs, laminar flow generally suppresses YAP/TAZ activity, whereas disturbed flow promotes their nuclear localization and transcriptional activity [56,57]. Beyond ECs, YAP/TAZ signaling is also essential for smooth muscle mechanobiology. Recent studies demonstrated that biomechanical stress activates adaptive transcriptional programs in thoracic aortic VSMCs that preserve aortic wall integrity and protect against aneurysm and dissection [58]. Consistent with this concept, VSMC-specific deletion of YAP/TAZ results in aneurysm formation, loss of contractile gene expression, proteoglycan accumulation, and inflammatory remodeling, supporting a critical role for YAP/TAZ in maintaining smooth muscle phenotype and aortic homeostasis [59]. These findings suggest that dysregulated YAP/TAZ signaling may contribute to aneurysm progression through effects on both endothelial adaptation to flow and smooth muscle responses to mechanical stress. In Marfan syndrome, impaired endothelial alignment to flow, altered junctional organization, and reduced eNOS/NO signaling contribute to oxidative stress, inflammation, and abnormal vascular tone regulation. NO signaling represents a major downstream effector of endothelial mechanotransduction and an important regulator of vascular homeostasis. Clinical studies demonstrating impaired flow-mediated and acetylcholine-induced vasodilatory responses in patients with Marfan syndrome provided some of the strong evidence of endothelial dysfunction in the disease [60,61,62]. Subsequent experimental studies confirmed reduced eNOS activity and diminished NO bioavailability in Marfan mouse models, supporting a role for impaired endothelial signaling in aneurysm pathogenesis [63,64,65]. Subsequent studies revealed additional layers of complexity in NO signaling within the aneurysmal aortic wall. While impaired eNOS activity and reduced NO bioavailability characterize endothelial dysfunction, increased expression of inducible nitric oxide synthase (iNOS/*NOS2*) has also been reported in Marfan aortas. iNOS-derived NO has been linked to activation of the NO–sGC–PKG signaling axis, smooth muscle dysfunction, and aneurysm progression [66,67]. Furthermore, accumulation of the ECM proteoglycan versican was recently shown to promote Akt-dependent Nos2 induction, providing a direct mechanistic link between ECM remodeling and altered NO signaling in Marfan syndrome [68]. In the setting of oxidative stress, excess NO may react with superoxide to generate peroxynitrite, thereby contributing to redox imbalance and tissue injury. In addition to vascular cells, inflammatory myeloid populations represent an important source of iNOS-derived NO, and NO signaling has been implicated in macrophage and neutrophil function, further linking nitrosative stress to the inflammatory component of HTAD pathogenesis [69].

Among the mechanosensors that transduce mechanical stimuli into biochemical responses in the vasculature are ion channels and G protein–coupled receptors, the apical glycocalyx, cytoskeletal networks, and the PECAM1–VE-cadherin–VEGFR2 junctional complex (reviewed in [70]). Of particular relevance to HTAD are stretch-activated angiotensin II type 1 receptor (AT1R) signaling and PIEZO1-mediated calcium influx. Although classically activated by angiotensin II, AT1R can also be activated directly by mechanical stretch [71]. In VSMCs, AT1R signaling contributes to adaptation to mechanical stress via Gq/11-PLC-Ca^2+^ signaling and stretch-induced ERK activation regulating vascular tone [72,73].

PIEZO1 is a shear-sensitive, Ca^2+^-permeable mechanosensitive ion channel expressed in ECs and required for normal vascular development and flow sensing [74,75]. In ECs, shear stress activates PIEZO1-dependent calcium influx and ATP release, triggering P2Y2–Gq/G11 signaling, eNOS activation, and NO production [76]. PIEZO1 also promotes KLF2/KLF4 expression through the Ca^2+^/CaMKII/MEKK3/ERK5 pathway to maintain endothelial homeostasis [77]. More recently, PIEZO1 has emerged as a direct modulator of Marfan aortopathy. PIEZO1 expression is reduced in the *Fbn1*^C1041G/+^ mouse model of Marfan syndrome, and conditional loss of PIEZO1 exacerbates thoracic aneurysm formation, inflammation, ECM remodeling, and TGF-β pathway activation through impaired endocytosis and autophagic degradation of TGFBR2 [78].

Although these pathways are often studied individually, substantial cross-talk exists among them. Mechanical activation of AT1R influences TGF-β signaling and ERK activation, while PIEZO1-dependent calcium influx regulates eNOS activity, NO bioavailability, and flow-responsive transcriptional programs. Likewise, alterations in TGF-β signaling can influence endothelial function, smooth muscle phenotype, and cellular metabolism, suggesting that mechanotransduction defects propagate through multiple interconnected signaling networks rather than through isolated pathways. Together, these observations support a model in which ECM abnormalities initiate coordinated disturbances in endothelial, smooth muscle, and fibroblast mechanosensing, ultimately driving maladaptive remodeling of the aortic wall.

## 5. Adventitial Remodeling and Fibroblast Heterogeneity in HTAD

While the medial layer has traditionally been considered the primary site of pathology in HTAD, increasing evidence suggests that the adventitia also contributes to disease progression. The adventitial ECM provides structural support to the aortic wall and has been proposed to function as an external mechanical scaffold that limits vessel expansion. Consistent with this concept, fibrillin-1 is not restricted to elastic fiber-associated microfibrils within the media but also forms independent microfibrillar networks in connective tissues, including the adventitia, suggesting that pathogenic variants affecting fibrillin biology may directly influence adventitial structure and function [79].

Recent advances in sc/snRNA-seq transcriptomics have further expanded our understanding of adventitial contributions to aortic disease. Fibroblasts exhibit substantial phenotypic heterogeneity rather than representing a homogeneous structural population, and undergo marked transcriptional reprogramming during aneurysm development. Studies in Loeys–Dietz syndrome mouse models identified distinct fibroblast populations characterized by ECM remodeling, inflammatory signaling, and stress-response programs, indicating active participation in disease progression rather than a purely supportive role [80,81]. These observations are consistent with emerging findings from human and experimental aneurysm datasets demonstrating that fibroblasts contribute to ECM turnover, intercellular signaling, and immune cell recruitment.

In addition to fibroblasts, the adventitial compartment contains the vasa vasorum, a specialized microvascular network that supplies oxygen and nutrients to the outer regions of the aortic wall and may also serve as a route for immune-cell trafficking. Studies in aneurysm disease have linked alterations in vasa vasorum structure and function to local hypoxia, neovascularization, endothelial activation, and inflammatory-cell infiltration, supporting the concept that adventitial microvascular remodeling may contribute to vessel wall degeneration and disease progression [82,83,84]. Although the precise contribution of vasa vasorum dysfunction to HTAD remains incompletely understood, these observations further support the concept that adventitial remodeling involves coordinated interactions among fibroblasts, immune cells, and vascular ECs rather than isolated changes in a single cellular compartment.

The recognition of fibroblast heterogeneity has important implications for understanding disease mechanisms. Together with ECs and VSMCs, fibroblasts represent a third major mechanosensitive cell population within the aortic wall capable of responding to altered ECM architecture and biomechanical stress. Moreover, their close association with infiltrating immune cells places the adventitia at the interface between structural remodeling and inflammation. These findings support a multicellular view of HTAD in which disease progression reflects coordinated dysfunction across all layers of the vessel wall rather than isolated abnormalities within the media alone.

## 6. Inflammation and Immune Cell Contributions in Thoracic Aortic Aneurysm

Although early studies linked progressive thoracic aortic aneurysm (TAA) formation in Marfan syndrome primarily to dysregulated TGF-β signaling, it is now clear that disease pathogenesis involves a more complex interplay of parallel and interacting cell types and pathways. Emerging work highlights critical contributions from EC–ECM mechanotransduction and inflammatory signaling, both of which represent potentially targetable processes in Marfan syndrome–associated cardiovascular disease [85,86,87,88,89].

In contrast to inherited aortic diseases, inflammation has long been considered a downstream consequence of acquired abdominal aortic aneurysm (AAA) disease [90]. However, accumulating evidence suggests that inflammatory processes play a more proximal and causative role in genetically mediated TAA. Notably, fibrillin-1 degradation products have been shown to promote macrophage chemotaxis [91], implicating matrix disruption as a direct initiator of immune cell recruitment. Supporting this concept, inhibition of macrophage recruitment, either through genetic inactivation of CC-chemokine receptor 2 or pharmacologic targeting of Jumonji domain-containing protein D3, attenuates aneurysm formation in murine models of both thoracic and abdominal aortic disease [88,92,93]. These findings underscore the importance of early immune cell trafficking in aneurysm pathogenesis and suggest that inflammation is not merely a secondary response.

Recent sc/snRNA-seq studies have substantially expanded this framework by revealing extensive immune-cell heterogeneity within the diseased aortic wall. In addition to inflammatory activation of ECs and VSMCs, these studies identified distinct macrophage, T-cell, and B-cell populations occupying predominantly adventitial and perivascular niches, highlighting complex immune–stromal interactions during aneurysm progression. In experimental TAAD, Liu et al. [94] identified an Il1rn^+^/Trem^1+^ macrophage population associated with disease progression, and pharmacologic inhibition of TREM1 reduced aortic rupture, supporting the concept that specific macrophage states actively contribute to disease evolution rather than simply responding to tissue injury. Human aortic dissection studies similarly demonstrated expansion of activated macrophage and T-cell populations, including Th17-like CD^4+^ T cells, together with enhanced macrophage–T-cell communication networks [95], suggesting that adaptive immune signaling contributes to the establishment of a chronic inflammatory microenvironment within the diseased aorta.

Beyond chemotaxis and matrix degradation, emerging evidence suggests that macrophage functional state may also influence tissue remodeling through interactions with neighboring stromal cells. Recent cardiovascular studies have shown that metabolic reprogramming of macrophages can promote fibroblast expansion and tissue fibrosis, raising the possibility that similar macrophage–fibroblast crosstalk may contribute to adventitial remodeling in HTAD [96]. Likewise, impaired efferocytosis, the process by which macrophages clear apoptotic cells, has emerged as an important regulator of inflammatory resolution and may be particularly relevant in aneurysmal tissues characterized by ongoing VSMC apoptosis and matrix degeneration [97]. Although these mechanisms have not yet been extensively investigated in HTAD, they represent potentially important avenues through which macrophage function could influence disease progression and chronic inflammation. Inflammatory signaling in TAA is not restricted to macrophages. Neutrophils have emerged as important contributors to acute aortic dissection through the release of proteases, reactive oxygen species, and neutrophil extracellular traps (NETs), which promote endothelial injury, ECM degradation, and medial degeneration [98]. Together, these findings support a model in which inflammatory responses arise through coordinated interactions among ECs, VSMCs, fibroblasts, macrophages, neutrophils, and lymphocytes across all layers of the aortic wall.

Translation of anti-inflammatory strategies into effective human therapies has yet been limited. In particular, doxycycline, a non-selective matrix metalloproteinase inhibitor, demonstrated efficacy in reducing aneurysm progression in rodent models of both TAA and AAA, yet failed to significantly alter aneurysm growth in randomized prospective human trials [99,100,101,102]. This disconnect likely reflects, in part, differences in disease stage at the time of intervention. In clinical settings, patients typically present with established aneurysms characterized by advanced ECM degradation and elastolysis, at which point targeting downstream inflammatory mediators may offer limited benefit. In addition, doxycycline has biological effects that extend beyond matrix metalloproteinase inhibition. As a tetracycline antibiotic, doxycycline can impair mitochondrial protein translation and oxidative phosphorylation, a mechanism increasingly recognized as contributing to its anti-inflammatory activity [103]. While suppression of mitochondrial metabolism may attenuate inflammatory cell activation, chronic inhibition of mitochondrial function could also adversely affect vascular cells already experiencing metabolic stress. Such pleiotropic effects may further complicate therapeutic responses and contribute to the challenges of translating promising preclinical anti-inflammatory strategies into effective clinical interventions.

Taken together, these observations highlight the critical importance of identifying the temporal context of therapeutic targeting. Our prior work demonstrates that modulation of TGF-β signaling in Marfan syndrome is highly stage-dependent and neutralization is deleterious early in disease development yet beneficial at more advanced stages [87]. A similar paradigm may apply to inflammatory pathways, wherein early immune cell recruitment represents a key driver of disease initiation, while later stages are dominated by irreversible structural degeneration. Mouse models provide a unique opportunity to define both mechanistic drivers and optimal therapeutic windows, which can then inform the design of human clinical trials.

Collectively, those studies, together with recent sc/snRNA-seq analyses, support an expanded framework for HTAD pathogenesis in which inflammation represents a third major axis, alongside endothelial dysfunction and maladaptive VSMC remodeling. Importantly, inflammatory responses are not restricted to infiltrating macrophages but involve coordinated interactions among fibroblasts, neutrophils, T cells, B cells, and vascular wall cells. The identification of distinct immune and stromal cell states further suggests that inflammation is a dynamic and stage-dependent process, reinforcing the need for temporally and cell type-specific therapeutic strategies.

## 7. Linking Genetic Variants to Cellular Pathways in HTAD

Early systematic analyses by Brownstein et al. [104] and subsequent ClinGen curation by Renard et al. [105] established that most HTAD-associated genes fall into three major functional categories: ECM components, the contractile apparatus, and intracellular signaling pathways (Table 1). This framework has remained consistent as additional genes, largely within these same categories, have been identified in more recent studies.

Among these genes, *FBN1* has been the most extensively studied. Work from Dietz and colleagues established that fibrillin-1 not only provides structural support but also regulates TGF-β bioavailability, linking ECM organization to growth factor signaling [23,106]. This concept was reinforced by studies of arterial biomechanics [19], which showed how matrix composition shapes the mechanical environment sensed by vascular cells.

Mutations in contractile genes such as *ACTA2* and *MYH11* disrupt pathways regulating cytoskeletal organization and force generation in smooth muscle cells [1,8]. These changes propagate through mechanosensitive systems that include integrin-associated adhesion complexes and caveolae. Caveolae were initially identified as key regulators of membrane mechanoprotection and signaling [107,108], and more recent work in the *Fbn1^C1041G/+^* mouse model of Marfan syndrome showed that *Cav1* influences endothelial and smooth muscle function, NO signaling, and aortic wall mechanics, negatively impacting TAAD progression [109].

Variants in signaling genes such as *TGFBR1*, *TGFBR2*, and *SMAD3* directly perturb TGF-β pathways that regulate ECM production, cellular differentiation, and inflammatory responses [11,110]. Importantly, disruption of TGF-β signaling within aortic smooth muscle cells can secondarily impair endothelial function. VSMC-specific loss of TGF-β signaling has been shown to increase vascular contractility and impair endothelium-dependent relaxation, linking altered smooth muscle signaling to reduced NO bioavailability and endothelial dysfunction [17].

Initial transcriptomic studies in Marfan syndrome used bulk RNA sequencing of aortic tissue from *FBN1* mutant mouse models, most commonly targeting the proximal/ascending aorta, the primary site of aneurysm development. These studies identified coordinated dysregulation of ECM remodeling, TGF-β–responsive transcriptional programs, and inflammatory signaling. In parallel, work from the Ramirez laboratory established AT1R signaling as a key pathogenic pathway, providing the rationale for therapeutic targeting with losartan [87].

Building on this framework, Galatioto et al. refined the role of AT1R by demonstrating that its pathogenic effects are cell-type specific. In *Fbn1*^mgR/mgR^ mice, endothelial-specific deletion of AT1aR improved survival, whereas VSMC-specific deletion did not confer comparable benefit [86]. These divergent outcomes were accompanied by distinct transcriptional responses, indicating that AT1R signaling engages different gene programs depending on the vascular compartment [86]. These findings establish that the contribution of a clinically relevant pathway cannot be understood at the tissue level alone, but depends critically on where within the vessel wall the signal is active, identifying the endothelium as a key driver of disease progression in this context.

Transcriptomic analyses have also enabled therapeutic discovery. Integration of gene expression signatures from mouse Marfan aorta and human Marfan vessel wall tissue identified repression of pathways related to smooth muscle contractility and cellular homeostasis, guiding drug repurposing strategies. This approach led to the identification of baclofen, and subsequent validation in *Fbn1*^mgR/mgR^ mice demonstrated improved aortic outcomes and restoration of transcriptional programs linked to contractile function [39]. Similarly, HIPK2 was identified as a regulator of disease-associated transcriptional responses, including fibrosis and stress signaling, and both genetic and pharmacological targeting was shown to attenuate aortic pathology in Marfan models [111]. Consistent with the growing utility of transcriptomic approaches for therapeutic target identification, more recent studies have implicated the PP2A–mTOR signaling axis in Marfan aortopathy. Pharmacological activation of PP2A attenuated aneurysm progression and partially normalized disease-associated transcriptional programs, further supporting the use of transcriptomics to identify actionable pathways linking vascular cell phenotype to disease progression [112].

More recent sc/snRNA-seq studies have refined these findings by assigning disease-associated transcriptional programs to specific vascular cell populations. In Marfan models and human Marfan aortic tissue, pathways initially identified in bulk analyses, including ECM remodeling, cytoskeletal dynamics, and inflammatory signaling, have been mapped to distinct but interacting populations of smooth muscle cells, ECs, and fibroblasts [31,33]. Together, these findings indicate that genetic defects in *FBN1* converge on shared pathway-level alterations that are executed in a cell-type–specific manner, providing a framework for more targeted therapeutic intervention.

Taken together, these observations support a model in which genetically diverse forms of thoracic aortic disease converge on interconnected pathways, ECM organization, cytoskeletal and contractile signaling, TGF-β signaling, and endothelial mechanotransduction and NO regulation, that govern cell–matrix communication. However, the relative contribution of each pathway varies by gene, cell type, and vascular context, indicating that aneurysm development arises from overlapping but non-uniform mechanisms and is unlikely to be addressed by a single therapeutic strategy across all forms of HTAD.

## 8. Current Pharmacological Therapy

Although the varied molecular and cellular mechanisms underlying different forms of HTAD are likely to be differentially important across genotypes, they converge at the level of vessel wall physiology, where dysregulated responses to biomechanical forces appear to be a major driver of disease. Accordingly, therapeutic efforts have focused primarily on reducing hemodynamic load through blood pressure control. Currently, no pharmacologic therapies are capable of reverting aortic aneurysms to a normal structural state or halting their progression; existing approaches aim to reduce the risk of aneurysm growth and rupture.

The most frequently used agents are beta-blockers, including metoprolol, propranolol, and atenolol, which reduce both blood pressure and heart rate. Reduction in heart rate is thought to decrease pulsatile wall stress and thereby reduce the risk of aneurysm progression. A foundational clinical study demonstrated approximately a three-fold reduction in aortic growth rate over ten years with propranolol treatment [113]. Angiotensin receptor blockers (ARBs) such as losartan are also used to attenuate aneurysm growth. A direct comparison of atenolol versus losartan in children and young adults over three years showed no significant difference between the two agents in inhibiting the rate of aortic enlargement [114]. More recently, an individual patient data meta-analysis of seven randomized trials involving 1442 patients with Marfan syndrome demonstrated that ARBs significantly reduced the rate of aortic root enlargement and that beta-blockers produced a similar magnitude of benefit, supporting the use of either class as standard medical therapy [115]. Importantly, the effect of ARBs was observed irrespective of background beta-blocker use, suggesting that the two therapies may provide complementary benefit when used in combination [115].

Although both beta-blockers and ARBs reduce blood pressure, they differ in their underlying mechanisms. The effects of beta-blockers are largely systemic, operating through hemodynamic regulation, whereas losartan additionally modulates TGF-β signaling, a pathway centrally dysregulated in Marfan syndrome. These mechanistic differences have driven considerable interest in ARBs as therapies capable of targeting disease-associated signaling in addition to reducing hemodynamic stress. In clinical practice, both classes are used either alone or in combination, with treatment decisions guided by patient characteristics, tolerance, and disease severity [3].

Calcium channel blockers are occasionally used when beta-blockers are not tolerated or contraindicated; however, their role in HTAD remains controversial. Experimental studies in Marfan mouse models demonstrated that certain calcium channel blockers, particularly dihydropyridines such as amlodipine, accelerate aneurysm progression and worsen survival, effects associated with enhanced ERK signaling and adverse aortic remodeling [116]. Importantly, retrospective clinical analyses have similarly suggested worse aortic outcomes in Marfan patients treated with calcium channel blockers compared with alternative antihypertensive regimens [116]. These findings raise concern that calcium signaling may have complex and context-dependent roles in aneurysm biology beyond blood pressure regulation.

Beyond currently used antihypertensive therapies, several emerging strategies seek to directly target pathogenic pathways implicated in aneurysm progression. Inhibition of mTOR signaling with rapamycin has been shown to attenuate aneurysm growth and improve survival in Marfan mouse models, supporting a role for metabolic and growth-regulatory pathways in disease progression [117]. Consistent with these observations, a recent study identified reduced PP2A activity as a contributor to Marfan aortopathy and demonstrated that pharmacological activation of PP2A with small molecule DT-061 attenuated aortic disease progression in two Marfan mouse models [112]. The beneficial effects were associated with suppression of mTOR signaling and preservation of smooth muscle cell contractile phenotype, identifying the PP2A–mTOR axis as a promising therapeutic target for future investigation [112]. Emerging evidence further suggests that mTOR-dependent signaling may intersect with RhoA/ROCK-mediated pathways involved in cytoskeletal organization and vascular remodeling. In a recently described HTAD model, pharmacological inhibition of ROCK attenuated thoracic aortic aneurysm and dissection, supporting a role for this pathway in disease progression and identifying ROCK signaling as a potential therapeutic target [118]. These findings are consistent with earlier studies in experimental AAA models, in which ROCK inhibition with fasudil reduced aneurysm formation and vascular remodeling [119]. Given the central role of RhoA/ROCK signaling in cytoskeletal tension, smooth muscle cell contractility, and mechanotransduction, this pathway represents an attractive target for modulating maladaptive biomechanical responses in HTAD. While these approaches remain experimental, they highlight a growing shift from hemodynamic control toward mechanism-based therapeutic intervention.

Finally, current agents slow aneurysm progression in Marfan syndrome, but they do not halt growth [4], underscoring the urgent need for new therapeutic strategies capable of stabilizing or reversing disease progression. Consistent with this need, early clinical studies continue to explore novel pathway-directed interventions. For example, a recent single-arm, open-label multicentre trial evaluated resveratrol treatment in adults with Marfan syndrome and observed stable aortic dimensions together with a trend toward reduced aortic growth rates at several thoracic aortic segments [120]. Although larger randomized studies will be required to establish clinical efficacy, these findings provide preliminary support for targeting disease-associated cellular pathways beyond conventional hemodynamic control.

## 9. Perspectives and Future Directions

Studies in mouse models of HTAD, particularly Marfan syndrome, have been valuable in defining key pathogenic pathways, including dysregulated TGF-β signaling, altered mechanotransduction, and impaired endothelial function. Importantly, these models have revealed that disease progression is not static but reflects dynamic, stage-dependent changes in cellular behavior and intercellular signaling. This has direct translational implications: interventions may be beneficial at one stage but ineffective or even detrimental at another, as demonstrated by the stage- and cell-specific effects of AT1R–TGF-β signaling in Marfan models [86,87]. It is equally important to develop a mechanistic understanding of the similarities and differences between disease progression in humans and in mouse models, as these distinctions will be critical for successful clinical translation. Comparative analyses of sc/snRNA-seq experiments can help identify the relevance of mouse model processes that may serve as drug targets to treat human disease.

The limited translational success of targeting AT1R signaling with losartan illustrates both the strengths and limitations of current approaches. While mouse model studies demonstrated robust effects on aortic aneurysm growth and signaling, clinical outcomes have been more modest, highlighting potential differences in the mechanisms underlying aneurysm progression in mouse and human systems, and suggesting that modulation of the AT1R pathway alone is not sufficient for a meaningful therapeutic response. These discrepancies likely reflect differences in timing, disease stage, and cell-type–specific pathway engagement. Similar considerations apply to emerging strategies informed by transcriptomic analyses, including drug repurposing approaches such as baclofen [39] and targeting of regulators such as HIPK2 [111], which show promise in preclinical models but require careful evaluation of their human relevance in the context of disease progression. For example, baclofen has complex systemic effects on blood pressure in humans, and its signaling effects on VSMCs would need to be distinguished from these hemodynamic actions to properly assess its therapeutic potential.

An additional complexity is highlighted by the paradoxical role of calcium signaling in HTAD. Although transcriptomic analyses in Marfan models have identified alterations in calcium handling and contractility-associated pathways, pharmacologic modulation of this axis has yielded unexpected results. Calcium channel blockers, despite their antihypertensive efficacy, have been associated with worsened aneurysm progression in both Marfan mouse models and retrospective patient studies [116]. These observations suggest that calcium signaling contributes to aneurysm biology through mechanisms extending beyond vascular tone regulation and emphasize the need for a more refined molecular understanding of calcium-dependent signaling pathways in disease-relevant vascular cell types before this axis can be rationally targeted therapeutically.

A central challenge moving forward is to define when and where pathogenic pathways and processes are active within the human aortic wall. Increasing evidence from genetic models and analyses of sc/snRNA-seq experiments indicate that ECs, VSMCs, fibroblasts, macrophages and other immune populations contribute differently over time, with shifts in cellular functional capabilities, metabolic state, and signaling networks accompanying aneurysm development [31,33,80,81]. These studies have fundamentally expanded the traditional view of HTAD as a disease driven primarily by medial degeneration, revealing instead a multicellular process involving coordinated changes across the intimal, medial, and adventitial compartments of the aortic wall. This underscores the need for longitudinal and cell-type–resolved approaches to map disease trajectories, rather than relying on single time-point analyses. The challenges of obtaining such data in human systems are substantial. Human aortic organoid models could offer a valuable path forward; while such systems do not yet exist in mature form, ongoing efforts to develop organoids incorporating VSMCs derived from disease-affected patients, matured under conditions of pulsatile flow, could provide powerful platforms for mechanistic study and drug discovery.

The identification of cell type-specific disease mechanisms also highlights the need for increasingly precise therapeutic strategies. Advances in targeted drug-delivery systems, including nanoparticle-based approaches, may eventually enable selective modulation of pathogenic pathways within specific vascular cell populations while minimizing systemic effects [121]. Likewise, continued progress in gene-editing and gene-replacement technologies raises the possibility of directly correcting causal genetic defects in HTAD [122]. Although these approaches remain largely experimental, they exemplify how improved mechanistic understanding may ultimately enable personalized and cell-specific therapeutic interventions.

Ultimately, translating mechanistic insight into effective therapies will require integrating genetic, transcriptomic, metabolic, and functional data to identify stage-specific and cell-type–specific intervention points. Recent advances in single-cell technologies have revealed that aneurysm progression is accompanied by dynamic changes in endothelial, smooth muscle, fibroblast, and immune-cell states, emphasizing that HTAD arises from coordinated dysfunction across the entire aortic wall rather than from abnormalities within a single cellular compartment. Therefore, future therapeutic strategies will need to account for the evolving nature of signaling and functional capabilities of multiple cell types across the aortic wall. Continued refinement of humanized animal models, coupled with human organoid and tissue-engineering approaches, will be essential to define therapeutic windows and develop interventions that stabilize the aorta before irreversible structural damage occurs.

## Figures and Tables

**Table 1:** Genes associated with heritable thoracic aortic disease (HTAD) and their primary pathways.

Gene	Syndrome/Disease	Functional Category	Primary Pathway/Process	Predominant Cell Type(s)
*FBN1*	Marfan syndrome	ECM component	Microfibril structure, TGF-β regulation, AT1R signaling	VSMC, EC
*TGFBR1*	Loeys–Dietz syndrome	Signaling	TGF-β receptor signaling	VSMC, EC
*TGFBR2*	Loeys–Dietz syndrome	Signaling	TGF-β receptor signaling	VSMC, EC
*SMAD2*	Loeys–Dietz–like	Signaling	TGF-β downstream signaling	VSMC
*SMAD3*	Aneurysm–osteoarthritis syndrome	Signaling	TGF-β transcriptional regulation	VSMC, fibroblast
*TGFB2*	Loeys–Dietz–like	Signaling	TGF-β ligand signaling	VSMC, EC
*TGFB3*	Loeys–Dietz–like	Signaling	TGF-β ligand signaling	VSMC
*ACTA2*	Familial TAAD	Contractile apparatus	Actin cytoskeleton, contraction	VSMC
*MYH11*	Familial TAAD	Contractile apparatus	Smooth muscle myosin function	VSMC
*MYLK*	Familial TAAD	Contractile apparatus	Myosin light chain phosphorylation	VSMC
*PRKG1*	Familial TAAD	Signaling	cGMP signaling/vascular tone	VSMC
*FLNA*	X-linked TAAD	Cytoskeleton	Actin crosslinking, mechanotransduction	VSMC
*COL3A1*	Vascular Ehlers–Danlos syndrome (vEDS)	ECM component	Collagen III structure	Fibroblast, VSMC
*COL5A1/COL5A2*	Classical EDS (occasionally vascular involvement)	ECM component	Collagen V assembly	Fibroblast
*EFEMP2 (Fibulin-4)*	Autosomal recessive cutis laxa	ECM component	Elastic fiber assembly	VSMC
*FBLN5*	Cutis laxa	ECM component	Elastic fiber assembly	VSMC
*LOX*	Familial TAAD	ECM remodeling	Collagen/elastin crosslinking	VSMC, fibroblast
*MAT2A*	Familial TAAD	Metabolic/epigenetic	Methylation/gene regulation	VSMC
*MFAP5*	Familial TAAD	ECM component	Microfibril-associated protein	VSMC
*BGN*	X-linked syndromic TAAD	ECM component	Proteoglycan/matrix organization	Fibroblast, VSMC

## Data Availability

No new data were created or analyzed in this study. Data sharing is not applicable to this article.

## Abbreviations

AAA	Abdominal Aortic Aneurysm
ARB	Angiotensin Receptor Blocker
AT1R	Angiotensin II Type 1 Receptor
EC	Endothelial Cell
ECM	Extra Cellular Matrix
Fbn1	Fibrillin1
HTAD	Heritable Thoracic Aortic Disease
MFS	Marfan syndrome
NO	Nitric Oxide
sc/snRNA-seqsingle	cell/single nuclei RNA sequencing
TGFβ	Transforming Growth Factor beta
TAA	Thoracic Aortic Aneurysm
VSMC	Vascular Smooth Muscle Cell
